# Analysis of Tacrolimus Clearance in Patients with Kidney Transplants from Romania

**DOI:** 10.3390/biomedicines13061501

**Published:** 2025-06-18

**Authors:** Corina Andreea Rotarescu, Ion Maruntelu, Ion Rotarescu, Alexandra-Elena Constantinescu, Ileana Constantinescu

**Affiliations:** 1 Department of Immunology and Transplant Immunology, “Carol Davila” University of Medicine and Pharmacy, 37 Dionisie Lupu Street, 020022 Bucharest, Romania; corina.rotarescu@drd.umfcd.ro (C.A.R.); alexandra-elena.constantinescu0720@stud.umfcd.ro (A.-E.C.); ileana.constantinescu@imunogenetica.ro (I.C.); 2 Department of Immunogenetics and Virology, Fundeni Clinical Institute, 258 Fundeni Avenue, 022328 Bucharest, Romania; 3 Department of Cardiovascular Surgery, Prof. Dr. C. C. Iliescu Emergency Institute for Cardiovascular Diseases, 258 Fundeni Avenue, 022328 Bucharest, Romania; rtrsc_i@yahoo.com; 4 Academy of Romanian Scientists (AOSR), 3 Ilfov Street, 030167 Bucharest, Romania

**Keywords:** tacrolimus, clearance, CYP3A4, kidney transplant

## Abstract

**Background/Objectives**: Tacrolimus is a key immunosuppressant in kidney transplantation, but its high interindividual pharmacokinetic variability complicates dosing. This study aimed to develop a population pharmacokinetic model and identify the factors explaining variability to optimize tacrolimus therapy in Romanian kidney transplant recipients. **Methods**: The study included 106 kidney transplant recipients treated at Fundeni Clinical Institute (2022–2024). Tacrolimus blood levels were measured using immunoassays, while gene polymorphisms of CYP3A4, CYP3A5, and ABCB1 were identified by real-time polymerase chain reaction. **Results**: Patients with CYP3A4*1/*1.001 impact clearance (RSE = 11.8%), while hematocrit was a significant covariate for intercompartmental clearance (RSE = 6.14%). **Conclusions**: Incorporating CYP3A4*1/*1.001 genotype and hematocrit into dosing strategies can improve therapeutic drug monitoring and personalize immunosuppressive therapy.

## 1. Introduction

### 1.1. General Background on Tacrolimus and Pharmacogenetics

Tacrolimus, a calcineurin inhibitor, is an essential immunosuppressive drug following kidney transplantation due to its ability to prevent graft rejection by suppressing T-cell activation. Tacrolimus is clinically effective but has a narrow therapeutic index and high interindividual pharmacokinetic variability. This variability requires accurate dose adjustments to achieve an optimal balance between therapeutic effect and the risk of toxicity or graft rejection [1,2,3,4]. The complexity in tacrolimus dosing highlights the need for individualized dosing strategies for kidney transplant recipients.

Pharmacogenetics plays an important role in understanding and addressing the variability in tacrolimus pharmacokinetics. Cytochrome P450 enzymes, especially CYP3A5 and CYP3A4, are the primary metabolizers of tacrolimus. Genetic polymorphisms within these enzymes, including the CYP3A5*1 allele, significantly influence the tacrolimus dose a patient requires. CYP3A5 expressers (carrying the *1 allele) require 40–50% higher doses than non-expressers (*3/*3 genotype) to achieve therapeutic levels [1,2,5,6,7]. Similarly, CYP3A4*1.001 (previously known as CYP3A4*1B) also contributes to variability in tacrolimus metabolism [8].

Variants in genes encoding drug transporters (e.g., *ABCB1*, *ABCC2*, *SLCO1B1*) also contribute to differences in tacrolimus absorption, distribution, and toxicity, though their clinical impact is less consistent and requires further study [6,9]. Incorporating pharmacogenetic information into dosing algorithms can enhance drug efficacy and safety [10,11,12].

### 1.2. Knowledge Gap in Romanian Kidney Transplant Recipients

Although pharmacogenetic-guided tacrolimus dosing is supported by evidence of efficacy in various populations, no data on Romanian kidney transplant recipients are available. This emphasizes the need for personalized immunosuppressant management in this population. This study uses a pharmacogenetics modelling approach to identify factors affecting tacrolimus dosing variability among Romanian kidney transplant patients.

## 2. Materials and Methods

### 2.1. Study Design and Patient Selection

This study involved 106 kidney transplant recipients treated at Fundeni Clinical Institute, Bucharest, Romania, between 2022 and 2024. Eligible patients were adults aged over 18 years who underwent kidney transplantation and were receiving tacrolimus-based immunosuppressive therapy within the first year after transplantation. Tacrolimus was administered orally every twelve hours according to the Fundeni Clinical Institute’s Nephrology Ward internal protocol. The initial dose ranged from 0.15 to 0.20 mg/kg/day, with subsequent adjustments to achieve target trough blood concentrations: 12–15 ng/mL in the first 14 days, 10–12 ng/mL during 15–30 days, 8–10 ng/mL during 31–60 days, and 6–8 ng/mL after 60 days.

Inclusion criteria required patients to have available demographic and laboratory data, including age, sex, weight, serum creatinine, and steady-state tacrolimus trough levels. Patients with incomplete medical records or significant comorbidities affecting drug metabolism (e.g., severe hepatic dysfunction or use of cytochrome P450 inhibitors) were excluded.

The Fundeni Clinical Institute Ethics Committee approved the study (No.46650/27.08.2024), which was conducted in accordance with the Helsinki Declaration. All subjects provided written informed consent to participate.

### 2.2. Genotyping and Drug Analysis

Blood samples (EDTA tubes) were collected at trough levels, immediately before the next dose, to determine tacrolimus trough concentrations (C0). Tacrolimus concentrations in whole blood samples were analyzed using a validated immunoassay (Device: Architech System^®^ i2000, and Kit: Architech Tacrolimus Assay, from Abbott (Abbott Diagnostic, Green Oaks, IL, USA). The daily dose of tacrolimus (Dose) was adjusted based on the patient’s weight and calculated as total daily dose divided by patient weight (mg/kg per day). The dose-adjusted tacrolimus trough concentration (C0/Dose ratio) was calculated by dividing the measured C0 value by the corresponding weight-adjusted daily dose (ng/mL per mg/kg per day).

A separate blood sample (EDTA tube) was collected for genotyping relevant pharmacogenetic markers: *ABCB1 C1236T* (rs1128503), *G2677T/A* (rs2032582), *C3435T* (rs1045642), CYP3A4*1.001 (rs2740574), CYP3A4*22 (rs35599367), and CYP3A5*3 (rs776746). DNA extraction was performed from 200 µL of whole blood using the QIAcube Instrument (Qiagen, Hilden, Germany) and the QiAmp DNA Blood Mini Kit (Qiagen, Hilden, Germany). Genotyping was conducted using the TaqMan^®^ Drug Metabolism Genotyping Assays from Life Technologies (Life Technologies Corporation, Pleasanton, CA, USA), which detect single-nucleotide polymorphisms (SNPs) [13]. The SNPs associated with genes encoding transport proteins and drug-metabolizing enzymes were identified using an Applied Biosystems 7300 Real-Time PCR System (Applied Biosystems—part of Thermo Fisher Scientific, Carlsbad, CA, USA).

### 2.3. Pharmacokinetic Modeling

Population pharmacokinetic analysis was performed using a nonlinear mixed-effects modelling approach implemented in Monolix 2019R2 (Lixoft, Switzerland, 2019). This analysis aimed to describe tacrolimus pharmacokinetics in the study population and identify significant covariates influencing its clearance.

Initially, linear or nonlinear elimination structural models were tested, including one-, two-, and three-compartment models. Residual variability was evaluated using constant, proportional, and combined error models. Model selection was guided by the decrease in objective function value (−2 × log-likelihood), the Akaike Information Criteria (AIC), the Bayesian Information Criteria (BIC), the parameter estimation expressed as the Relative Standard Error (RSE%), Goodness-of-Fit (GOF) plots, and Visual Predictive Checks (VPC).

#### 2.3.1. Covariate Testing

After establishing the base model, continuous and categorical covariates were tested for their effects on primary pharmacokinetic parameters: clearance (Cl), central volume of distribution (V1), intercompartmental clearance (Q), and peripheral volume of distribution (V2). The continuous covariates were age and hematocrit, while categorical covariates were modelled as binary factors. Covariates showing a significant trend were further evaluated using a stepwise regression approach with the −2 log-likelihood value, and significant covariates were incorporated into the final model.

#### 2.3.2. Model Evaluation

GOF plots were utilized as the initial assessment of model suitability. VPC was conducted to evaluate the performance of the final model.

The final model parameters simulated tacrolimus concentration-time profiles under various dosing regimens. Monte Carlo simulations (normal size 10,000) were performed to predict the probability of achieving therapeutic target concentrations while avoiding toxicity. The simulation results informed recommendations for personalized dosing in the study population.

### 2.4. Statistical Analysis

Statistical analyses were performed using IBM SPSS version 20 and GraphPad Prism version 9.3.0. In SPSS software, we introduced demographic, clinical, and genetic variables, such as age, weight, sex, *ABCB1*, *CYP3A4*, and *CYP3A5* genotypes. All administered doses, all measured concentrations, and all biological parameter values for each patient (estimated glomerular filtration rate (eGFR) (Cockcroft-Gault formula), alanine aminotransferase (ALT), aspartate aminotransferase (AST), albumin, hematocrit) to account for inter-patient variability, were also included in the statistical analysis.

The potential impact of CYP3A4*1.001, CYP3A4*22, CYP3A5*3, *ABCB1 C3435T*, *ABCB1 C1236T*, and *ABCB1 G2677T/A* genotypes on C0, Dose, and C0/Dose ratio of tacrolimus was estimated using the Mann–Whitney U test and Kruskal-Wallis test as appropriate. Additionally, the Mann–Whitney U test was used to study the impact of different genetic combinations on tacrolimus C0. *p*-values < 0.05 were considered statistically significant after being adjusted using the Bonferroni correction.

All independent variables were analyzed for their association with tacrolimus concentrations. We performed a stepwise linear regression, including genotypes and biochemical parameters significantly associated with tacrolimus concentration. The obtained independent variables with *p*-values < 0.05 were then used for population modelling.

## 3. Results

### 3.1. Study Population

The study included patients over 40 years old (53.77%) and was predominantly male (63.2%). The entire study group’s key demographic and clinical characteristics are summarized in Table 1. Overall, hematologic parameters and liver function tests (ALT and AST) were within the normal range. The mean tacrolimus C0 was 10.08 ± 4.36 ng/mL, achieved with a mean weight-adjusted daily dose of 0.127 ± 0.889 mg/kg.

Genotype analysis revealed a predominance of the *CYP3A4* wild-type/wild-type (wt/wt) genotype (85.8%) and *CYP3A5* mutant/mutant (m/m) genotype (76.4%) (see Table 2).

### 3.2. Association of SNP Genotypes with C0, Dose, and C0/Dose Ratio of Tacrolimus

Patients were grouped based on their genotypes for each genetic variant we evaluated. Each group represents a subset of the study population, defined only by the presence or absence of specific genetic variants.

The mean C0, dose, and C0/Dose ratios of tacrolimus in renal transplant recipients were grouped based on their *CYP3A4*, *CYP3A5*, and *ABCB1* genotypes to assess the relationship between genetic polymorphisms and tacrolimus pharmacokinetics (Table 3). Pairwise comparisons between genotype groups were performed using either the Mann–Whitney U test or the Kruskal-Wallis test, as appropriate.

The study identified significant differences in tacrolimus clearance between genotypes. Specifically, patients with the CYP3A4*1/*1 genotype exhibited higher tacrolimus concentrations and a greater C0/Dose ratio compared to those with the *1/*1.001 genotype (*p* < 0.05).

Similarly, patients carrying the CYP3A5*3/*3 genotype showed significantly higher C0 values and required lower tacrolimus doses than those with the *1/*3 genotype (*p* < 0.05). The C0/Dose ratio was also significantly higher in the *3/*3 group (*p* < 0.05).

**Figure 1:**
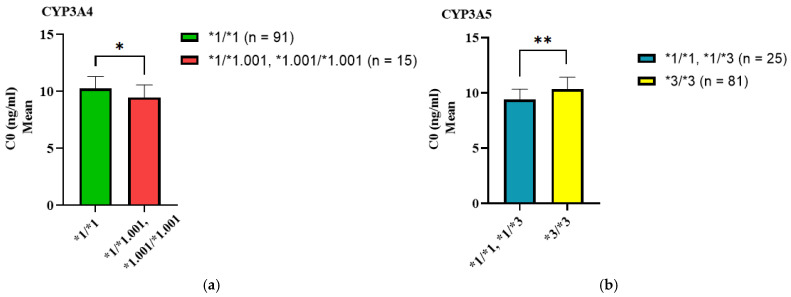
The effect of CYP3A4*1.001 and CYP3A5*3 genotypes on C0 of tacrolimus. (**a**) The absence of CYP3A4*1.001/*1.001 significantly decreases the tacrolimus C0 in the investigated cohort. (**b**) The combined presence of CYP3A5*1/*1 and CYP3A5*1/*3 in the studied cohort significantly reduced tacrolimus C0. *p*-values calculated with the Mann-Whitney U test: ** *p* < 0.001; * *p* ≤ 0.05; C0: trough concentration.

We analyzed the impact of *CYP3A4* and *CYP3A5* genotypes on tacrolimus C0 levels. Individuals carrying at least one CYP3A4*1.001 allele had 1.07-fold lower tacrolimus C0 levels than homozygous CYP3A4*1/*1 carriers. Likewise, carriers of at least one active CYP3A5*1 allele (CYP3A5*1/*1 or *1/*3) exhibited 1.08-fold lower tacrolimus C0 levels compared to CYP3A5*3/*3 homozygotes (see Table 4, Figure 1).

### 3.3. Tacrolimus Clearance

We further investigated *CYP3A4* and *CYP3A5* genotypes in a stepwise multiple linear regression after showing their impact on tacrolimus C0 in univariate analysis. Additionally, factors like sex, weight, age, hematocrit, albumin, eGFR, ALT and AST were tested individually. Factors achieving *p*-values < 0.05 were included in a stepwise multiple linear regression model. After backward selection, the final model consisted of CYP3A4*1.001, CYP3A5*3, age and hematocrit. Tacrolimus C0 interindividual variability was largely explained by the model (all *p* < 0.05) (see Table 5).

To investigate variability in tacrolimus C0, we applied a two-compartment population pharmacokinetic model with first-order absorption, lag time, and linear elimination. This model estimated key pharmacokinetic parameters—absorption, clearance, and distribution—and identified covariates (factors) contributing to interindividual variability. Among these, the CYP3A4*1/*1.001 genotype and hematocrit level were significant predictors.

The model’s beta coefficient (beta_Cl_CYP3A4*1/*1.001) shows that typical clearance is approximately 23% lower in patients with the *1/*1.001 genotype compared to the patients with other genotypes. Clinically, this finding is important. It predicts higher blood levels for a given dose in these patients, which helps explain individual dosing requirements. Higher hematocrit levels were also associated with reduced intercompartmental clearance, reflecting altered drug distribution between the bloodstream and tissues.

Including the previously mentioned covariates made the model more accurate at predicting individual tacrolimus levels. Table 6 presents the estimated parameters for drug handling processes, quantifies the impact of these covariates, and shows the remaining variability.

Table 6 data showed that the final model’s lag time (Tlag) was 0.541 h (RSE 9.46%), which indicates a comparatively short delay before absorption starts. A rapid absorption phase was also indicated by the absorption rate constant (ka), which was found to be 4.03 h⁻¹ (RSE 17%).

Interestingly, the similarity in Tlag and ka values between the base and final models suggests that including covariates primarily affected the drug’s disposition and elimination rather than the absorption process. Introducing the CYP3A4*1.001 genotype as a covariate demonstrated a moderate yet significant impact on clearance.

The final model estimated Cl at 0.759 L/h (RSE 7.36%) and revealed a negative coefficient for the genotype term (beta_Cl_CYP3A4*1/*1.001 = −0.259; RSE 11.8%). This suggests the importance of inter-individual genetic variability in tacrolimus metabolism and that subjects with such a genotype may need more careful dose optimization.

Additionally, the volumes of distribution in the central and peripheral compartments (V1 and V2) showed slight increases in the final model, recorded at 0.396 L and 39.1 L, respectively.

The effect of hematocrit on intercompartmental clearance (beta_Q_Hematocrit = −0.0382; RSE 6.14%) suggests that higher hematocrit levels may reduce the rate at which tacrolimus redistributes between compartments. This finding is consistent with physiological predictions that changes in blood composition can significantly impact the dynamics of drug distribution.

Additionally, for most parameters, interindividual variability (omega) remained moderate (see Table 7). Individual differences in tacrolimus metabolism were highlighted by the estimated variability in clearance (omega_Cl) of 0.393 (RSE 12.3%).

Combining these insights and adding covariates enhanced the model’s overall fit and improved its dose individualization in patients to achieve therapeutic targets while minimizing the risk of toxicity.

**Figure 2:**
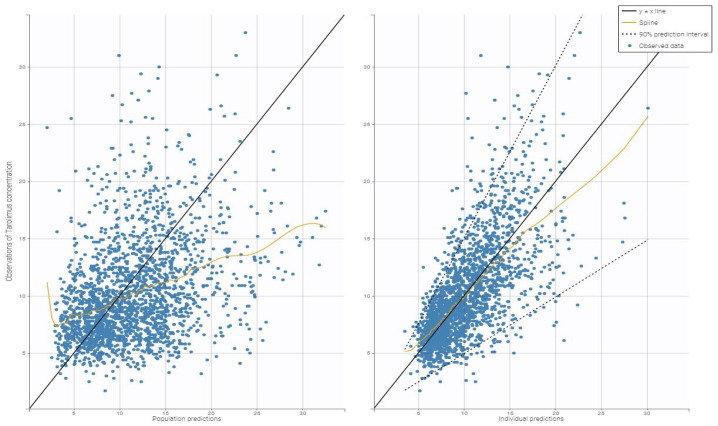
Goodness-of-fit plots for the final population pharmacokinetic model. The left plot shows population predictions of tacrolimus concentrations compared to observed values, while the right plot displays individual predictions of tacrolimus versus the observed concentrations.

Figure 2 shows the diagnostic plots for the tacrolimus final covariate model, which confirm the correctness of parameter calculations.

**Figure 3:**
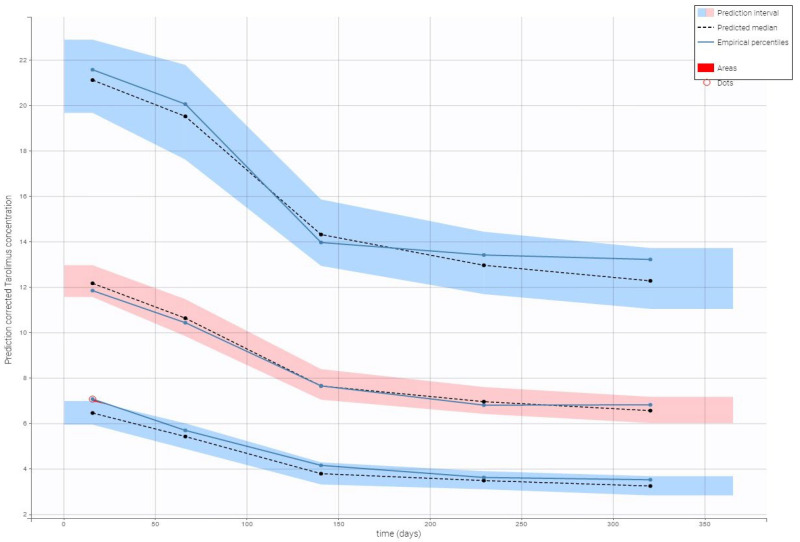
Prediction-corrected visual predictive check for the final model of tacrolimus. A visual predictive check (VPC) for the final tacrolimus model illustrates model performance against observed data. Observed tacrolimus concentrations (circles; ng/mL) are plotted along with blue lines representing their 10th, 50th, and 90th percentiles. Simulated confidence intervals for the percentiles are shown as shaded regions (red for the median, blue for the 5th and 95th simulated bounds). Observed points highlighted in red are identified as outliers.

Visual predictive check plots confirmed that the final model effectively captured the observed data, with predicted concentrations falling within the 90% prediction interval. As shown in Figure 3, the strong agreement between observed and simulated data supports the model’s reliability in predicting tacrolimus pharmacokinetics.

## 4. Discussion

Genetic factors significantly influence the pharmacokinetic variability of tacrolimus, leading to various pharmacogenetic models for predicting starting doses [14,15,16,17,18]. Because the patient’s *CYP3A* and *ABCB1* genotype is often unknown when prescribing, pharmacokinetic models integrating genetic, demographic, and clinical covariates offer a more accurate approach to predicting pre-transplant clearance, improving post-transplant outcomes. Although some models integrate these factors, few have been prospectively validated [14,15,16,17,18]. Studies such as those by Andrews et al. and Francke et al. have shown that algorithm-guided dosing is more effective in achieving target therapeutic ranges than weight-based methods [19,20]. However, an important gap remains: specific populations remain underrepresented in these analyses (e.g., Eastern Europeans). By building our population model, the present study contributes to optimizing tacrolimus dosing in our study population (Romanian renal transplant patients), emphasizing the need for personalized treatment strategies.

### 4.1. Key Findings

The impact of CYP3A4*1.001 and CYP3A5*3 polymorphisms on tacrolimus pharmacokinetics in recipients of renal transplants from Romania was assessed in this study.

We found that CYP3A4*1/*1.001 genotype carriers, identified as intermediate metabolizers (15.3% of participants), presented significantly higher blood trough concentrations and C0/Dose ratio compared to non-carriers, suggesting reduced clearance. Also, in 76% of participants, carriers of the CYP3A5*3/*3 genotype, we observed higher tacrolimus C0 and C0/Dose ratio than those with the CYP3A5*1/*3 genotype. These results support the role of the CYP3A5*3 allele in reducing tacrolimus metabolism, leading to higher plasma concentrations and increased systemic exposure. Another key result of our model was the demonstration of the impact of covariates: the model that included the CYP3A4*1/*1.001 polymorphism and pre-transplant hematocrit predicted an increased clearance (0.759 L/h versus 0.564 L/h without these covariates), highlighting the importance of these factors in explaining the pharmacokinetic variability in the study population.

### 4.2. Comparison with Literature

To place these key findings in the context of the existing literature, we compared our results on the impact of *CYP3A4* and *CYP3A5* polymorphisms on tacrolimus pharmacokinetics. They are generally consistent with previous studies, although there are also notable differences, such as those reported by Concha et al. [21]. In contrast to our results indicating reduced clearance in CYP3A4*1/*1.001 carriers, the study by Concha et al. [21] found that patients with at least one CYP3A4*1.001 allele required a higher dose and had lower blood concentrations and dose-adjusted levels. This difference could be related to population or methodological characteristics.

Regarding *CYP3A5*, both studies emphasize the importance of the non-functional CYP3A5*3 allele, which was the majority in our cohort. The biological mechanism [22,23,24] explains why carriers of the homozygous CYP3A5*3/*3 genotype metabolize tacrolimus more slowly. Concha et al. [21] raise an important issue regarding the linkage disequilibrium between CYP3A5*3 and CYP3A4*1.001 and the potential collinearity in multivariable models. This statistical aspect may influence which of the two factors is retained in the model, explaining why some studies, including that of Concha et al., chose to exclude CYP3A5*3/*3 from their final model, to reduce uncertainty in estimates and avoid overlapping genetic effects [21,24].

### 4.3. Clinical Implications

These findings have direct clinical implications for the optimization of individualized tacrolimus therapy. Our data highlight the importance of considering CYP3A4*1.001 genotype and pre-transplant hematocrit to guide dosing in the early post-transplant period, as patients with the CYP3A4*1/*1.001 genotype and/or higher hematocrit values have slower drug clearance. For these patients, an initial dose reduction of 15–25% from the conventional dose of 0.15–0.20 mg/kg/day is warranted in clinical practice (e.g., 0.12–0.13 mg/kg/day for a threshold of 0.15 mg/kg/day). In cases with significantly elevated hematocrit, it is prudent to increase monitoring to ensure adequate tacrolimus exposure even when using a dose of 0.15 mg/kg/day. For patients without this genotype and with normal hematocrit, an initial dose of 0.15–0.20 mg/kg/day remains recommended, with subsequent adjustments based on the target trough concentration.

Although genotype and hematocrit improve dosing accuracy, therapeutic drug monitoring (TDM) remains essential to verify that trough concentrations are maintained within the recommended range (e.g., 12–15 ng/mL during the first two weeks). Monitoring in the early days post-transplant may be influenced by complex individual characteristics and rapid changes in metabolism, such as acute variations in liver function or emerging drug interactions. Additional individual factors, such as concomitant medications and changes in clinical status, should also inform subsequent dose adjustments. The proposed model could support a personalized approach to immunosuppressive therapy among Romanian patients after it is validated in a large cohort.

### 4.4. Limitations

Despite our findings, the study also has limitations. The relatively high standard error for some fixed effects (e.g., beta_Cl_CYP3A4*1/*1.001: 11.8%) suggests that additional data could increase the precision of these estimates. The absence of validation data from other hospitals limits the model’s generalizability to larger populations. Future studies should include external validation of the model in other centres in Romania or similar populations.

## 5. Conclusions

This study developed a population pharmacokinetic model for tacrolimus in Romanian renal transplant patients. The model highlighted the importance of the CYP3A4*1.001 genotype and pre-transplant hematocrit as critical factors for optimizing the initial tacrolimus dosing before transplantation. By integrating these factors, we can create a personalized approach that effectively targets the therapeutic range while minimizing the risks of early overexposure or underexposure. To implement these findings in clinical practice—potentially through guided dosing tools—it is essential to validate the model externally in diverse patient populations. This validation will ensure that the results are generalizable and confirm the benefits on a broader scale.

## Figures and Tables

**Table 1:** Patient Demographics and Clinical Characteristics.

Patient Parameters	Mean ± Standard Deviation
Age	
18–39 years	29.94 ± 6.41
40–66 years	48.04 ± 5.98
Sex (male/female)	67/39
Weight (kg)	68.05 ± 14.65
Hematocrit (%)	35.38 ± 7.32
Albumin (g/dL)	44.4 ± 0.54
eGFR (mL/min/1.73 m^2^)	45.18 ± 18.78
ALT (U/L)	31.63 ± 25.43
AST (U/L)	23.21 ± 10.26
Tacrolimus C0 (ng/mL)	10.08 ± 4.36
Tacrolimus Dose (mg/kg per day)	0.127 ± 0.889
Tacrolimus C0/Dose (ng/mL/mg/kg per day)	115.88 ± 97.52

eGFR = estimated glomerular filtration rate; ALT = alanine aminotransferase; AST = aspartate aminotransferase; Tacrolimus C0 = trough concentration of tacrolimus. The values are presented as descriptive statistics (mean ± standard deviation or frequencies).

**Table 2:** Genotype Distribution.

Genotype (n = 106)	wt/wt	wt/m	m/m
CYP3A4*1.001	91 (85.8%)	15 (14.2%)	0 (0%)
CYP3A5*3	7 (6.6%)	18 (17%)	81 (76.4%)
CYP3A4*22	24 (22.6%)	82 (77.4%)	0 (0%)
*ABCB1 3435C>T*	23 (21.7%)	53 (50%)	30 (28.3%)
*ABCB1 1236C>T*	22 (20.8%)	47 (44.3%)	37 (34.9%)
*ABCB1 2677G>T/A*	35 (33%)	40 (37.7%) (*G/A*) 2 (1.9%) (*G/T*)	27 (25.5%) (*A/A*) 2 (1.9%) (*T/A*) 0 (0%) (*T/T*)

wt = wild-type; m = mutant. The values are presented as descriptive statistics (frequencies and percentages).

**Table 3:** Impact of Genotype on Mean Tacrolimus Concentration (C0), Dose and C0/Dose Ratio.

Genes	SNP/Allele	N	Tacrolimus Concentration (C0)	*p*-Value	Dose	*p*-Value	C0/Dose Ratio	*p*-Value
CYP3A4*1.001	*1/*1	91	10.17 ± 4.42	0.041 *	0.124 ± 0.088	0.075	120.5 ± 101.6	0.047
	*1/*1.001	15	9.46 ± 3.86		0.150 ± 0.906		86.58 ± 57.93	
	*1.001/*1.001	0						
CYP3A5*3	*1/*1	7	9.67 ± 3.58	0.002 *	0.131 ± 0.076	0.016 *	91.14 ± 44.10	0.012 *
	*1/*3	18	9.31 ± 3.97		0.162 ± 0.093		79.91 ± 58.61	
	*3/*3	81	10.28 ± 4.48		0.120 ± 0.087		125.7 ± 105.1	
CYP3A4*22	*1/*1 (n = 24)	24	9.72 ± 4.16	0.084	0.124 ± 0.085	0.565	116.45 ± 94.30	0.728
	*1/*22 (n = 82)	82	10.19 ± 4.41		0.129 ± 0.089		115.70 ± 98.55	
	*22/*22 (n = 0)	0						
*ABCB1 3435C>T*	CC (n = 23)	23	10.06 ± 4.35	0.766	0.121 ± 0.081	0.569	122.02 ± 82.48	0.419
	CT (n = 53)	53	10.11 ± 4.35		0.127 ± 0.079		111.42 ± 101.08	
	TT (n = 30)	30	10.02 ± 4.38		0.135 ± 0.105		118.27 ± 101.89	
*ABCB1 1236C>T*	CC (n = 22)	22	10.24 ± 4.49	0.788	0.111 ± 0.071	0.410	126.68 ± 80.93	0.318
	CT (n = 47)	47	9.94 ± 4.15		0.124 ± 0.079		116.92 ± 109.18	
	TT (n = 37)	37	10.14 ± 4.51		0.142 ± 0.105		108.38 ± 90.75	
*ABCB1 2677G>T/A*	GG (n = 35)	35	10.11 ± 4.56	0.710	0.132 ± 0.098	0.973	112.65 ± 92.25	0.971
	GA (n = 40)	40	10.05 ± 4.22		0.123 ± 0.080		123.55 ± 115.53	
	AA (n = 27)	27	10.03 ± 4.32		0.120 ± 0.077		111.34 ± 77.34	

*p*-value for comparing the two genotypes derived from the Mann-Whitney U test. *p*-value for comparing the three genotypes derived from the Kruskal–Wallis test. Data are shown as mean ± standard deviation. Only significant *p*-values that remained significant after Bonferroni adjustment are shown as corrected *p*-values, marked with an asterisk (*). *ABCB1 2677GT/TA/TT* genotypes were excluded from the analysis because very few patients (n = 4) have these genotypes.

**Table 4:** Combined Analysis of CYP3A4 and CYP3A5 Genotypes on Tacrolimus Concentration.

Genes	SNP/Allele	N	Tacrolimus Concentration (C0)	*p*-Value
CYP3A4*1.001	*1/*1	91	10.17 ± 4.42	0.041 *
	*1/*1.001 *1.001/*1.001	15	9.46 ± 3.86	
CYP3A5*3	*1/*1 *1/*3	25	9.49 ± 3.77	0.0004 *
	*3/*3	81	10.28 ± 4.48	

*p*-value for comparing the genotype models derived from the Mann–Whitney U test; data shown as mean ± standard deviation. Only significant *p*-values that remained significant after Bonferroni adjustment are shown as corrected *p*-values, marked with an asterisk (*).

**Table 5:** Linear Stepwise Regression Analysis.

Variable	Partial r^2^	*p*-Value
CYP3A4*1.001	0.301	**<0.001**
CYP3A5*3	0.297	**<0.001**
Age	0.304	**0.004**
Hematocrit	0.285	**<0.001**

Significant differences are marked in bold.

**Table 6:** Fixed Effects Parameter Estimates and Their Standard Errors.

Parameter	Base Model			Final Model		
	Estimate	Standard Error (SE)	Relative Standard Error (RSE%)	Estimate	Standard Error (SE)	Relative Standard Error (RSE%)
Fixed Effects						
Tlag (h)	0.547	0.0638	11.7	0.541	0.0512	9.46
ka (h^−1^)	3.79	0.974	25.7	4.03	0.684	17
Cl (L/h)	0.564	0.0341	6.04	0.759	0.0559	7.36
beta_Cl_CYP3A4*1/*1.001	-	-	-	−0.259	0.0307	11.8
V1 (L)	0.341	0.11	32.2	0.396	0.0452	11.4
Q (L/h)	0.72	0.0705	9.79	2.26	0.227	10
beta_Q_Hematocrit	-	-	-	−0.0382	0.00235	6.14
V2 (L)	43.2	7.54	17.4	39.1	5.4	13.8

Explanatory Notes: Tlag (h): the estimated delayed time before the drug is absorbed into the bloodstream (e.g., 0.541 h). It helps to understand how long it will take for the drug to enter the patient’s system; ka (h^−1^): estimated rate of drug absorption. It affects how quickly the drug concentration rises and reaches its peak level; Cl (L/h): the estimated rate at which the body removes the drug from the central circulation (clearance). This is a primary determinant of overall drug exposure and is important for determining appropriate dosing rates and frequencies to maintain therapeutic levels; beta_Cl_CYP3A4*1/*1.001: estimated effect of the CYP3A4*1/*1.001 genotype on clearance. This identifies a genetic factor that predicts slower drug removal, which might mean these patients need lower doses to avoid high drug levels; V1 (L): estimated initial space the drug distributes into immediately after entering the bloodstream (central volume). This influences the initial drug concentration achieved, which is important for loading doses or understanding immediate post-administration levels; Q (L/h): the estimated rate at which the drug moves between the central blood circulation and other tissues (intercompartmental clearance). This describes how quickly and extensively the drug distributes throughout the body; beta_Q_Hematocrit: estimated effect of hematocrit levels on how the drug distributes between blood and tissues (Q). This shows that hematocrit levels are estimated to influence how the drug spreads into tissues; V2 (L): estimated additional space (volume) the drug distributes outside the central circulation (peripheral volume). Together with V1, it defines the amount of space occupied by the drug in the body, influencing drug concentrations over longer periods; RSE (%): Relative Standard Error. Calculated as (Standard Error/Estimate) × 100%. This percentage (shown in the RSE columns) indicates how confident the model’s estimated value is based on the data. It provides insight into the reliability of the numbers presented. Low RSE (e.g., <20–30%): The estimate is considered reliable and well-supported by the data. It shows that a clinical practitioner can have reasonable confidence in using this value for clinical interpretation or prediction.

**Table 7:** Random Effects Parameter Estimates and Their Standard Errors.

Parameter	Base Model			Final Model		
	Estimate	Standard Error (SE)	Relative Standard Error (RSE%)	Estimate	Standard Error (SE)	Relative Standard Error (RSE%)
**Standard Deviation of the Random Effects**						
omega_Tlag	0.13	0.109	84	0.239	0.0497	20.8
omega_ka	0.256	0.121	47.4	0.557	0.375	67.4
omega_Cl	0.395	0.0461	11.7	0.393	0.0482	12.3
omega_V1	1.12	0.196	17.4	0.632	0.0511	8.09
omega_Q	0.3	0.0599	20	0.38	0.0426	11.2
omega_V2	1.46	0.182	12.5	1.16	0.105	9.08
**Error Model Parameters**						
b	0.306	0.0053	1.73	0.307	0.00523	1.7
**−2 × log-likelihood**	11,859.63			11,856.36		
**AIC**	11,893.63			11,882.36		
**BIC**	11,938.91			11,916.99		

Explanatory Notes: omega_Tlag to omega_V2: These represent the standard deviations of interindividual variability for the respective parameters, indicating how much individual responses differ from the population average for each parameter; b: residual unexplained variability–accounts for the deviation between observed and predicted concentrations not explained by the model. A lower value indicates the model does a good job of predicting the actual drug levels measured in patients, suggesting the model fits the individual data points well; −2 × log-likelihood (−2LL): a goodness-of-fit measure. Lower values indicate a better-fitting model; AIC (Akaike Information Criteria): penalizes model complexity to avoid overfitting. Lower values suggest a more optimal balance of fit and parsimony. Lower values for AIC (comparing the Final Model to the Base Model) indicate a better fit to the data; BIC (Bayesian Information Criteria): similar to AIC, but with a stronger penalty for the number of parameters. Lower values for BIC (comparing the Final Model to the Base Model) indicate a better fit to the data; A model that fits the data better is considered a more reliable description of how the drug behaves in the patient population.

## Data Availability

The dataset is available on request from the authors.
